# Driver mutations associated with signatures of platinum sensitivity in germ cell tumors

**DOI:** 10.1038/s41698-024-00727-2

**Published:** 2024-11-02

**Authors:** Yun Cheng Sawa, Liwei Jia, Harris Krause, Margaret Meagher, Frederick Millard, Andrew Elliott, John T. Lafin, Christina Jamieson, Emmanuel S. Antonarakis, Anishka D’Souza, Krinio Giannikou, James F. Amatruda, Siamak Daneshmand, Rana R. McKay, Matthew Oberley, Chadi Nabhan, Aditya Bagrodia

**Affiliations:** 1 Department of Urology, University of California San Diego, La Jolla, CA USA; 2 Department of Pathology, University of Texas Southwestern Medical Center, Dallas, TX USA; 3 Caris Life Sciences, Irving, TX USA; 4 University of Minnesota, Minneapolis, MN USA; 5 Cancer and Blood Disease Institute, Children’s Hospital Los Angeles, Los Angeles, CA USA; 6 Departments of Paediatrics and Medicine, Keck School of Medicine, University of Southern California, Los Angeles, CA USA; 7 Department of Medicine, University of California San Diego, La Jolla, CA USA; 8 Department of Urology, University of Texas Southwestern Medical Center, Dallas, TX USA

**Keywords:** Tumour heterogeneity, Cancer genomics

## Abstract

We sought to evaluate the genomic and transcriptomic landscapes in primary and metastatic germ cell tumors (GCTs; *N* = 138) to uncover factors that drive cisplatin resistance. Prevalence was calculated for platinum-resistant alterations (PRAs; *KRAS*, *TP53*, and *KIT* mutations, and *MDM2* amplification) and high copy number amplifications (CNA ≥ 6 copies). Tumors were designated as chemo-naïve (PreC, *N* = 66) or post-chemotherapy (PostC, *N* = 17). A transcriptomic signature associated with platinum sensitivity (PSS, high suggests increased sensitivity) was applied. *KIT* mutations were observed in 14.5% of primary versus 1.8% of met and 0% of lymph. *TP53* mutations were identified in 10% of primary GCTs versus 17% of met and 16.7% of lymph. *MDM2* CNAs were similar between sites. PRA-positive PreC GCTs had significantly lower average PSS scores compared to PRA-negative tumors. Lower PSS scores in chemo-naïve tumors were associated with PRAs, suggesting a potential mechanism for platinum resistance.

## Introduction

Germ cell tumors (GCTs) are neoplasms that arise from germ cells, traditionally stratified based on anatomical locale into gonadal and extragonadal tumors^1^. Ninety-five percent of GCTs are testicular in origin^2–4^. Over the past few decades, the incidence of testicular GCTs (TGCTs), predominantly seminomas, has risen, with approximately 9190 new cases and 470 deaths from testicular cancer anticipated in the United States in 2023^5^. Given that testicular GCTs are fifteen times more prevalent than other GCTs, the bulk of data to date dissecting the molecular underpinning of disease pathogenesis is derived from studies profiling tumors derived from the testis^4^.

The mainstay of treatment for GCTs is surgical extirpation of the primary site, followed by observation, chemotherapy, or radiation therapy, depending on tumor type and stage. Cisplatin-based chemotherapy regimens are highly efficacious in the treatment of GCTs, with cure rates above 95% for most patients^4^. Despite this high cure rate, a subset of patients develop cisplatin resistance, leading to disease recurrence and poor outcomes^4,6^. We hypothesize that the genomic and transcriptomic landscapes of primary and metastatic GCTs may reveal driver factors of cisplatin resistance and organotropism in GCTs.

Molecular hallmarks of testicular GCTs, including low tumor mutational burden (TMB), presence of isochromosome 12p (i12p), and high prevalence of *KIT* and *KRAS* mutations, are well described^3^. More recent analyses indicate that pathogenic mutations and copy number alterations (CNAs) in primary and metastatic sites in GCTs are highly heterogenous^7^. This information underscores the genomic complexity inherent in the progression of GCTs, which may influence chemotherapy response and patient outcomes. Prior work by our group suggests that TP53 pathway alterations, including *TP53* mutations (primarily in mediastinal nonseminoma GCTs) and *MDM2* amplification, are associated with chemotherapy resistance. Nevertheless, molecular underpinnings of platinum resistance and organotropism in GCTs are incompletely understood.

In this study, we utilized a large database of GCT patient samples that underwent genomic and transcriptomic sequencing to elucidate genetic alterations underlying organotropism and cisplatin resistance in GCTs.

## Methods

### Cohort selection

The study cohort included GCTs (*N* = 138) that underwent comprehensive molecular profiling between 2010 and 2022 at Caris Life Sciences (Phoenix, AZ) (Table 1).

**Table 1:** Patient demographic of the study cohort subgroups stratified by biopsy site include primary tumors (Primary), non-lymph node metastases (Met), and lymph node metastases (Lymph)

	Primary	Met	Lymph	Statistic	*q*-Value
Count (*N*)	65	59	14		
Median age [range] (N)	27 [6 - 65] (65)	34 [14 - 82] (59)	41 [27 - 56] (14)	Kruskal–Wallis	<0.001
Female	29.2% (19/65)	13.6% (8/59)	7.1% (1/14)	Chi-square	0.042
% Chemo naïve	92.1% (35/38)	73.5 (25/34)	54.5% (6/11)	Chi-square	0.013
*Primary tumor site*					
Ovary	24.6% (16/65)	10.2% (6/59)	7.1% (1/14)	Fisher’s Exact	0.034
Testicle	55.4% (36/65)	74.6% (44/59)	57.1% (8/14)		
Suprasellar	3.1% (2/65)	0.0% (0/59)	0.0% (0/14)		
Descended testis	1.5% (1/65)	1.7% (1/59)	0.0% (0/14)		
Mediastinum	7.7% (5/65)	3.4% (2/59)	14.3% (2/14)		
Brain, NOS	4.6% (3/65)	0.0% (0/59)	7.1% (1/14)		
Pineal gland	1.5% (1/65)	0.0% (0/59)	0.0% (0/14)		
Pituitary gland	1.5% (1/65)	0.0% (0/59)	0.0% (0/14)		
Misc.	0.0% (0/65)	10.1% (6/59)	14.3% (2/14)		

This study was conducted in accordance with the guidelines of the Declaration of Helsinki, the Belmont Report, and the US Common Rule. In keeping with 45 CFR 46.101 (b), this study was performed utilizing retrospective, de-identified clinical data. Therefore, this study was deemed Institutional Review Board exempt, and no patient consent was necessary from the subjects.

### Histopathologic features to determine the status of chemotherapy and tumor sites

**Fig. 1:**
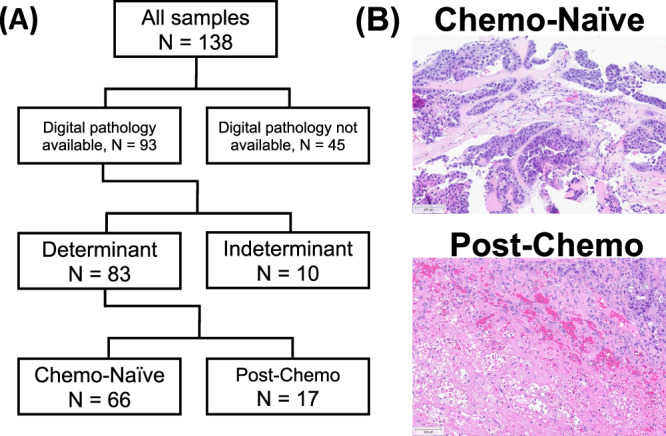
Classification of pathologic samples. **A** Schematic of how tumors were defined as “Chemotherapy-Naïve” or “Post-Chemotherapy” via pathology review. **B** Representative H&E stains for Chemo Naïve and Post-chemo tumors.

A board-certified genitourinary pathologist (L.J.) centrally reviewed the available digitalized hematoxylin and eosin (H&E)-stained slides from 93 GCTs. A clear determination of chemotherapy status was feasible for 83 tumors based on the presence of areas of ghost outlines of the necrotic cells, surrounding fibrosis, inflammatory cell infiltrate and/or xanthomatous/fibroxanthomatous reaction. These tumors were further classified as chemo-naïve (*N* = 66) or post-chemo (*N* = 17) (Fig. 1). A primary tumor (*N* = 65) was defined as a tumor collected from the annotated primary anatomic site. A metastatic (non-lymph) tumor (*N* = 59, met) was defined as any non-primary tumor sampled from non-lymph node anatomic sites. A lymph metastasis (*N* = 14, lymph) was defined as any tumor obtained from any lymph node.

### DNA next-generation sequencing (NGS)

A targeted 592-gene panel or whole exome sequencing (WES) was performed using genomic DNA isolated from formalin-fixed paraffin-embedded (FFPE) tumor tissue. The 592-gene pane (Supplementary Table 1) was sequenced using the NextSeq platform (Illumina, Inc., San Diego, CA). A custom-designed SureSelect XT assay was used to enrich 592 whole-gene targets (Agilent Technologies, Santa Clara, CA). WES was performed using the manufacturer’s guidelines and the Illumina NovaSeq 6000 sequencer (Illumina, Inc). A hybrid pull-down panel of baits was designed to enrich 719 clinically relevant genes (Supplementary Table 2) with an average sequencing depth of coverage of >500, along with another panel designed to enrich additional >20,000 genes at a lower depth. The performance of the 592-gene and WES assays was validated for sequencing variants and copy number amplification events (high amplification was considered >6 copies: -hAmp). Briefly, a concordance study comparing the WES panel to Caris’ previously validated test NGS-592 panel included 113 samples that spanned 18 different lineages, covered a wide range of tumor cell density (20–90% tumor nuclei) and variants frequency (8–100%). Additionally, a concordance study comparing WES to an independently validated WES Assay performed at TGen, included 65 samples out of the 113 listed above, which spanned 16 different lineages and covered a wide range of tumor cells density (30% - 90% tumor nuclei) and variant frequency (8–94%). Both studies found that assays were highly concordant. DNA from matched normal tissue was not available for sequencing this GCT cohort.

### Classification of genetic variants

Genetic somatic variants identified were interpreted by board-certified molecular geneticists and categorized as ‘pathogenic,’ ‘likely pathogenic,’ ‘variant of unknown significance,’ ‘likely benign,’ or ‘benign,’ according to the American College of Medical Genetics and Genomics (ACMG) standards and using the following databases: COSMIC, UCSC Genome Brower, PubMed, HGMD, gnomAD database, ClinVar database, dbSNP database, InSiGHT database, IARC TP53 database, LOVD databases, BRCA Exchange, GeneReviews, Atlas of Genetics and Cytogenetics in Oncology and Hematology, CIViC database, cBioPortal, OMIM database, COSMIC Fusions If a variant has an rs number associated with it the dbSNP database must also be consulted to determine minor allele frequency of the mutation and based on the ‘Standards and Guidelines for the Interpretation of Sequence Variants: A Joint Consensus Recommendation of the American College of Medical Genetics and Genomics and the Association for Molecular Pathology’. When assessing mutation frequencies of individual genes, ‘pathogenic,’ and ‘likely pathogenic’ were counted as mutations while ‘benign’, ‘likely benign’ variants and ‘variants of unknown significance’ (VUS) were excluded unless otherwise noted.

### Whole transcriptome RNA-sequencing

FFPE specimens underwent pathology review to diagnose percent tumor content and tumor size; a minimum of 10% of tumor content in the area for microdissection was required to enable enrichment and extraction of tumor-specific RNA. A Qiagen RNA FFPE kit (Qiagen, Germantown, MD, USA) was used, and the RNA quality and quantity were determined using the Agilent TapeStation (Agilent Technologies). Biotinylated RNA baits were hybridized to the synthesized and purified cDNA targets, and the bait-target complexes were amplified in a post-capture PCR reaction. The resultant libraries were quantified and normalized, and the pooled libraries were denatured, diluted, and sequenced. For transcript counting, transcripts per million molecules (TPM) were generated using the Salmon expression pipeline.

### Immune signatures

Immune cell fractions of ten different cell types were calculated using quanTIseq, an immune deconvolution algorithm that utilizes RNA transcripts known to be expressed in specific immune cell types to deconvolute bulk RNA sequencing data and predict the different immune cell fraction present in the bulk RNA lysate^8^. WTS data was also used to calculate a T cell-inflamed score as previously described^9^.

### Immunohistochemistry (IHC)

IHC was performed on FFPE sections of glass slides. Slides were stained using automated staining techniques, per the manufacturer’s instructions, and were optimized and validated according to CLIA/CAP and ISO requirements. Positive staining of PD-L1 (SP142) was defined as ≥2+ of the intensity and ≥5% positive stain.

### Microsatellite instability/mismatch repair deficiency (MSI-H/dMMR) status

A combination of multiple test platforms was used to determine MSI-H/dMMR status of the tumors profiled, including immunohistochemistry (IHC) of mismatch repair proteins (MLH1, M1 antibody; MSH2, G2191129 antibody; MSH6, 44 antibodies; and PMS2, EPR3947 antibody [Ventana Medical Systems, Inc., Tucson, AZ, USA]) and NGS (for tumors tested with NextSeq platform, >2800 target microsatellite loci were examined and compared to the reference genome GRCh37 (hg19). The two platforms generated highly concordant results as previously reported, and in the rare cases of discordant results, the MSI-H or MMR status of the tumor was determined by IHC^10^.

### Tumor mutational burden (TMB)

TMB was measured by counting all non-synonymous missense, nonsense, in-frame insertion/deletion, and frameshift mutations found per tumor that had not been previously described as germline alterations in dbSNP151, Genome Aggregation Database (gnomAD) or benign variants identified by Caris geneticists. A cutoff point of $\ge$10 mutations per megabase (MB) was used based on the KEYNOTE-158 pembrolizumab trial, which showed that patients with a TMB of ≥10 mt/MB (TMB-H) across several tumor types had higher response rates than patients with a TMB of <10 mt/MB^11,12^.

### Platinum sensitivity score and platinum resistance alterations

A previously published gene set of 23 genes whose expression was either positively or negatively associated with platinum resistance (in ovarian cancer) was used to estimate a platinum sensitivity score (PSS)^13^. Gene expression values (TPM) were *z*-score normalized. *Z*-score values were added if associated with chemosensitivity and subtracted if associated with resistance to create a PSS. A high PSS is associated with more sensitivity to platinum therapy, and a lower score is associated with more resistance. Platinum resistance alterations (PRAs) and driver mutations included *KRAS*, *TP53*, and *KIT* mutations, and *MDM2* amplification. Additionally, based on our data, any tumors with *KRAS* amplification were considered PRA-negative, as tumors with these mutations tended to have higher PSS scores (Supplementary Fig. 2).

### Statistical methods

Statistical analyses were conducted utilizing Mann–Whitney *U* (scipy V.1.9.3) and *X*^2^/Fisher-Exact tests (R v.3.6.1) for continuous and categorical variables, respectively. *P*-values were adjusted for multiple comparisons using the Bonferroni correction, with *p* < 0.05 considered statistically significant.

## Results

### Patient cohort

Sixty-five primary tumors were sequenced, including 7 intracranial primary tumors, 5 mediastinal primary tumors, 16 ovarian primary tumors, and 37 testicular primary tumors. Seventy-three samples were obtained from metastatic sites (including lymph nodes) based on clinician annotation. This included two from bone, 11 from the brain, six from the liver, five from the lung, 14 from lymph nodes, four from the mediastinum, 12 from the peritoneum, one from the spermatic cord, and 18 from non-bone/liver/brain visceral sites (kidney, small intestine, connective tissue, etc). Compared to non-lymph node and lymph node metastases, patients with biopsy from primary tumors had a significantly lower median age at the time of biopsy (24 vs 34 and 41 years, respectively, *p* < 0.001), were more frequently female (29.2% vs 13.6% and 7.1%, respectively, *p* < 0.42), and were more frequently chemo-naïve (92.1% vs 73.5% and 54.5%, respectively, *p* < 0.01) (Table 1).

### The genomic and transcriptomic landscape of primary and metastatic GCTs

**Fig. 2:**
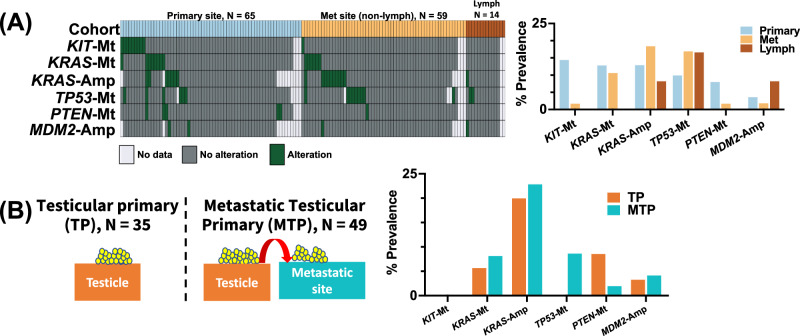
Alterations by tumor site. **A** Oncoprint and prevalence of alterations (Copy number amplifications [Amp] and pathogenic SNV/indel [Mt] between Primary, non-lymph metastatic and lymph metastatic tumors. **B** Occurrence of alterations between testicular primary and metastatic testicular primary tumors.

The genomic variation landscape of GCTs was sparse, and predominantly made up of recurrent genetic variants previously associated with driver mutation or chemotherapy resistance (*KIT*-Mt, *KRAS*-Mt, *TP53*-Mt*, PTEN*-Mt, *KRAS*-Amp, and *MDM2*-Amp). As a result, our investigation focused on these genes (Fig. 2A). Notably, *KIT*-Mt was observed in 14.5% of primary cases versus 1.8% in met and 0% in lymph (*p* = 0.76). *TP53*-Mt was identified in 10% of primary GCT versus 17% in met and 16.7% in lymph (*p* > 0.99). Primary, met, and lymph cohorts had similar prevalence of *KRAS*-Amp (13% vs 18.5% vs 8.3%, *p* > 0.99) and *MDM2*-Amp (3.7% vs 1.9% vs 8.3%, *p* > 0.99). *KRAS*-Mt prevalence was similar between primary (12.9%) and met (10.7%) but was lower in the lymph (0.0%) cohort (*p* > 0.99). No mutations were significantly enriched in any of the investigated cohorts. Sub-setting our cohort to tumors of testicular origin, no significant difference in alteration prevalence was observed between testicular primary (TP, *N* = 35) and metastatic testicular primary (MTP: tumors whose primary site is testicular and whose specimen site is metastatic [no longer testicular]), *N* = 49) GCTs (*p* > 0.05). However, *TP53*-Mt was exclusively observed in the MTP cohort (8.7% vs 0%, *p* > 0.05). There were no significant differences between primary tumors and metastatic tumors from mediastinal mediastinum, ovarian, or intracranial tumors either (Supplementary Fig. 1). Notably, 100% of mediastinal primary tumors harbored *TP53* mutations.

### Comparative analysis between post-chemotherapy and chemo-naïve GCTs

**Fig. 3:**
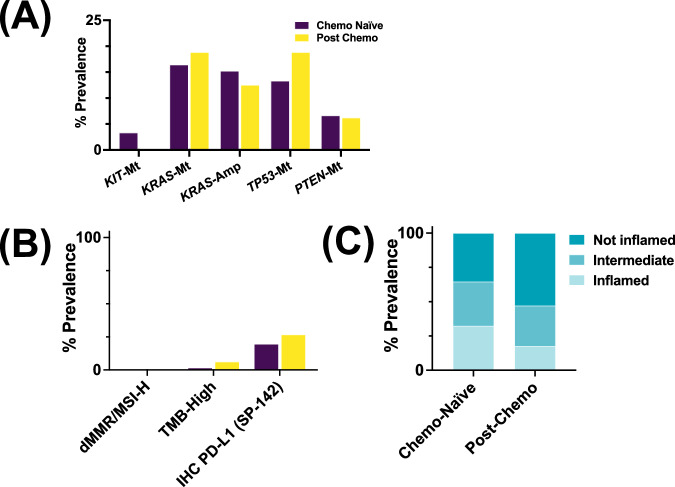
Comparison of chemotherapy-naive and post-chemotherapy tumors. **A** Prevalence of alterations, **B** immune checkpoint inhibitor biomarkers, and **C** T cell-inflamed tumors in Chemo-Naïve vs-Post Chemo tumors.

No significant differences in the prevalence of platinum resistance associated-genetic variants were observed between chemo-naïve and post-chemo GCTs (*p* > 0.05) (Fig. 3A). There were no MMRd/MSI-H in PostC nor PreC GCTs. Although not significant, postC tumors had a slightly higher prevalence of TMB-H (6.2% vs 1.6%) and PD-L1+ (26.7% vs 19.6%) compared to the PreC group (*p* > 0.98 each). PreC tumors had a higher fraction of T cell-inflamed tumors compared to PostC tumors (32% vs 18%, *p* = 0.35) (Fig. 3C), although this did not reach significance.

### Evaluating a gene expression signature associated with platinum resistance

**Fig. 4:**
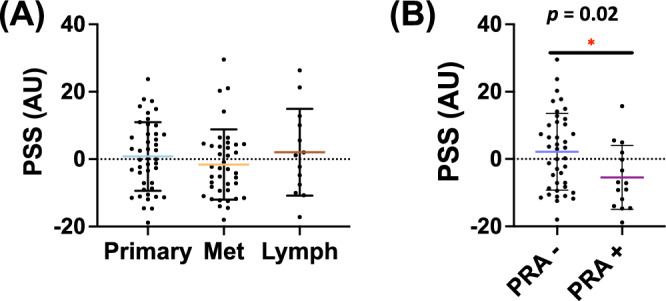
Platinum sensitivity scores. **A** Mean platinum sensitivity score (PSS) between primary, non-lymph metastatic, and lymph metastatic tumors (*p* > 0.05) (arbitrary units: AU). **B** Mean PSS score in chemo naïve tumors ± platinum-resistant alteration (defined as Mt-*KRAS*, Mt-*TP53*, Mt-*KIT*, or CNA*-MDM2*. Any tumors with CNA-*KRAS* alteration were not considered as part of the PRA group as tumors with these mutations tended to have higher PSS scores).

In order to define gene signatures related to platinum resistance, a transcriptomic score made up of genes associated with platinum sensitivity was applied. Platinum sensitivity scores were similar between primary, met, and lymph tumors (0.81 vs −1.60 vs 2.06 AU, *p* > 0.05) (Supplementary Fig. 2). PreC GCTs harboring platinum resistance alterations (PRA+) had an average PSS of −5.44 AU, while PreC PRA-tumors had an average PSS of 2.17 AU (*p* = 0.02) (Fig. 4B). KRAS-Amp alterations were often concurrent with another PRA and were generally associated with a positive PSS (Supplementary Fig. 1), which may suggest a potential link between MAPK signaling and platinum sensitivity. Additionally, although rare, both MDM2-Amp tumors had very low PSS (Supplementary Fig. 1).

## Discussion

The development of cisplatin resistance in GCTs remains incompletely understood. Utilizing a cohort of both primary and metastatic GCTs, we characterized the genomic landscape of GCTs. We did not identify recurrent molecular features driving organotropism or platinum resistance. The prevalence of platinum resistance alterations did not vary by primary or metastatic site. We did identify a subset of tumors that may be predicted to be platinum-sensitive using a gene signature associated with platinum sensitivity.

Previous research has investigated the genomic landscape of GCTs and identified mutations likely to contribute to oncogenesis, platinum resistance, and metastatic potential. In a study of 664 TGCTs, Loveday et al., identified seven molecular hallmarks of TGCT tumorigenesis, including association of genetic variants with metastatic potential^14^. These results were reproduced in our cohort, with the identification of *KRAS*, *TP53*, and *KIT* mutations and *MDM2* amplification in GCTs associated with gene expression related to cisplatin resistance. Our group previously reported the contribution of *TP53*-Mt in GCTs with cisplatin resistance^3^. With regards to the impact of *TP53*-Mt on metastatic progression, subsequent studies conducted by Timmerman et al. revealed that *TP53*-Mt frequently occurs in tumors localized to the mediastinum^15^. The current study further corroborates the above findings, as *TP53*-Mt was identified exclusively in the MTP cohort as compared to the TP cohort. Indeed, in the present study, *TP53* mutations were identified in all mediastinal primary tumors. Though the occurrence of somatic *KIT* mutations is primarily observed in seminomas, its role in tumorigenesis and invasion remains inconclusive^16,17^. Although not statistically significant, our finding that primary GCTs had a higher prevalence of *KIT* mutations offers a potentially therapeutic target and prognostic marker, warranting further investigation.

A transcriptomic signature associated with platinum resistance was applied to identify the relationship between genetic alterations and treatment sensitivity. In chemotherapy naïve tumors, a set of mutations (PRA+) was shown to be associated with lower platinum sensitivity, whereas PRA negativity conferred a higher platinum sensitivity score. Additional validation of our PSS is needed in germ cell tumors, but after substantial validation, this score could potentially be applied prior to initiation of platinum therapy and might help identify patients who could benefit from treatment escalation or de-escalation.

The increased expression of PD-L1 in TGCTs compared to normal testicular tissue noted in prior studies has provided the rationale for exploring the role of immunotherapy in TGCTs^18,19^. Several clinical trials have investigated anti-PD-L1 agents but have been terminated prematurely due to lack of efficacy^20–22^. In our cohort, chemotherapy naïve patients were more frequently classified as “T-cell inflamed,” which is associated with a more positive response to immunotherapy^12^. Interestingly, tumors labeled as post-chemo were associated with a less inflamed state. This perhaps underlies a mechanism whereby receipt of chemotherapy selects for a microenvironment in which immunotherapy is unlikely to render benefit and perhaps explains the lack of efficacy noted in the above clinical trials. Future trials may consider the addition of immunotherapy to standard chemotherapeutic regimens for patients with poor-risk features.

Our genomic and transcriptomic analysis did not reveal recurrent molecular alterations that explain the poor prognosis among patients who harbor non-pulmonary, visceral metastases. While enrichment of *TP53* mutations among mediastinal primary tumors may explain relative platinum insensitivity, the complex interplay between tumor microenvironment, organotropism, and impact on platinum sensitivity remains to be elucidated. A prior study focusing on paired primary and metastatic GCT samples elucidated high rates of heterogeneity among samples from varying sites, for both copy number changes and somatic mutations, which may also partially explain our results^8^.

Our study is limited by the inherent biases introduced in a retrospective study design. Additionally, clinical follow-up of patients with the sequenced tumors in this study was not available. While pre- vs post-chemotherapy sample state could be reliably determined by histopathological examination, the actual clinical status of the patient (chemotherapy sensitive versus resistant) was not available. Despite these limitations, our findings suggest that chemo-naïve GCTs may harbor platinum resistance alterations that have the potential to modulate the efficacy of subsequent platinum-based treatment. Additional prospective and retrospective studies are required to validate this signature and investigate its clinical utility.

In conclusion, a higher prevalence of *TP53*-Mt was identified in metastatic GCTs, whereas *KIT*-Mt was more common in the primary cohort, with a subset of alterations shown to be associated with platinum resistance-related gene expression. Overall, these findings present compelling evidence that genetic alterations may serve as determinants of cisplatin resistance. If validated in GCTs. further studies, our findings might be used as potential biomarkers to guide therapeutic decisions and improve the management of patients with GCTs by enabling early detection of tumors likely to harbor platinum resistance.

## Supplementary information

Supplemental Material

## Data Availability

The datasets analyzed during the current study are not publicly available but are available from the corresponding author on reasonable request (AB).
