# Supplementary material for: Driver mutations associated with signatures of platinum sensitivity in germ cell tumors
Source: NPJ Precis Oncol. 2024 Nov 2;8:249. doi: 10.1038/s41698-024-00727-2 (PMC11531533; doi:10.1038/s41698-024-00727-2)

Supplementary Table 1 ABI1 (10p12.1)

ABL1 (9q34.12) ABL2 (1q25.2) ACKR3 (2q37.3) ACSL3 (2q36.1) ACSL6 (5q31.1) ADGRA2 (8p11.23) AFDN (6q27) AFF1 (4q21.3-22.1) AFF3 (2q11.2) AFF4 (5q31.1) AKAP9 (7q21.2) AKT1 (14q32.33) AKT2 (19q13.2) AKT3 (1q43-44) ALDH2 (12q24.12) ALK (2p23.2-23.1) AMER1 (Xq11.2) APC (5q22.2)

AR (Xq12)

ARAF (Xp11.3) ARFRP1 (20q13.33) ARHGAP26 (5q31.3) ARHGEF12 (11q23.3) ARID1A (1p36.11) ARID2 (12q12) ARNT (1q21.3) ASPSCR1 (17q25.3) ASXL1 (20q11.21) ATF1 (12q13.12) ATIC (2q35)

ATM (11q22.3) ATP1A1 (1p13.1) ATP2B3 (Xq28) ATR (3q23) ATRX (Xq21.1) AURKA (20q13.2)

AURKB (17p13.1) AXIN1 (16p13.3) AXL (19q13.2) BAP1 (3p21.1) BARD1 (2q35) BCL10 (1p22.3) BCL11A (2p16.1) BCL11B (14q32.2) BCL2 (18q21.33) BCL2L11 (2q13) BCL2L2 (14q11.2) BCL3 (19q13.32) BCL6 (3q27.3)

Gene

BCL7A (12q24.31) BCL9 (1q21.2) BCOR (Xp11.4) BCORL1 (Xq26.1) BCR (22q11.23) BIRC3 (11q22.2) BLM (15q26.1) BMPR1A (10q23.2) BRAF (7q34) BRCA1 (17q21.31) BRCA2 (13q13.1) BRD3 (9q34.2) BRD4 (19p13.12) BRIP1 (17q23.2) BTG1 (12q21.33) BTK (Xq22.1) BUB1B (15q15.1) C15orf65 (15q21.3) CACNA1D (3p21.1) CALR (19p13.13)

CAMTA1 (1p36.31-

36.23)

CANT1 (17q25.3) CARD11 (7p22.2)

CARS1 (11p15.4) CASP8 (2q33.1) CBFA2T3 (16q24.3) CBFB (16q22.1) CBL (11q23.3) CBLB (3q13.11) CBLC (19q13.32) CCDC6 (10q21.2) CCN6 (6q21) CCNB1IP1 (14q11.2) CCND1 (11q13.3) CCND2 (12p13.32) CCND3 (6p21.1) CCNE1 (19q12) CD274 (9p24.1) CD74 (5q33.1) CD79A (19q13.2) CD79B (17q23.3) CDC73 (1q31.2) CDH1 (16q22.1) CDH11 (16q21) CDK12 (17q12) CDK4 (12q14.1) CDK6 (7q21.2) CDK8 (13q12.13) CDKN1B (12p13.1) CDKN2A (9p21.3) CDKN2B (9p21.3) CDKN2C (1p32.3) CDX2 (13q12.2) CEBPA (19q13.11) CHCHD7 (8q12.1) CHEK1 (11q24.2) CHEK2 (22q12.1) CHIC2 (4q12)

CHN1 (2q31.1)

CIC (19q13.2) CIITA (16p13.13)

CLP1 (11q12.1) CLTC (17q23.1) CLTCL1 (22q11.21) CNBP (3q21.3) CNOT3 (19q13.42) CNTRL (9q33.2) COL1A1 (17q21.33) COPB1 (11p15.2) COX6C (8q22.2) CREB1 (2q33.3) CREB3L1 (11p11.2) CREB3L2 (7q33) CREBBP (16p13.3) CRKL (22q11.21) CRLF2 (Xp22.33) CRTC1 (19p13.11) CRTC3 (15q26.1) CSF1R (5q32) CSF3R (1p34.3) CTCF (16q22.1) CTLA4 (2q33.2) CTNNA1 (5q31.2) CTNNB1 (3p22.1) CYLD (16q12.1) CYP2D6 (22q13.2) DAXX (6p21.32) DDB2 (11p11.2) DDIT3 (12q13.3) DDR2 (1q23.3) DDX10 (11q22.3) DDX5 (17q23.3) DDX6 (11q23.3) DEK (6p22.3) DICER1 (14q32.13) DNM2 (19p13.2) DNMT3A (2p23.3) DOT1L (19p13.3) EBF1 (5q33.3)

ECT2L (6q24.1) EGFR (7p11.2) EIF4A2 (3q27.3) ELF4 (Xq26.1) ELK4 (1q32.1) ELL (19p13.11) ELN (7q11.23) EML4 (2p21) EMSY (11q13.5) EP300 (22q13.2) EPHA3 (3p11.1)

EPHA5 (4q13.1-13.2) EPHB1 (3q22.2) EPS15 (1p32.3) ERBB2 (17q12) ERBB3 (12q13.2) ERBB4 (2q34)

ERC1 (12p13.33) ERCC1 (19q13.32) ERCC2 (19q13.32) ERCC3 (2q14.3) ERCC4 (16p13.12) ERCC5 (13q33.1) ERG (21q22.2) ESR1 (6q25.1-25.2) ETV1 (7p21.2) ETV4 (17q21.31) ETV5 (3q27.2) ETV6 (12p13.2) EWSR1 (22q12.2) EXT1 (8q24.11) EXT2 (11p11.2) EZH2 (7q36.1) EZR (6q25.3) FANCA (16q24.3) FANCC (9q22.32) FANCD2 (3p25.3) FANCE (6p21.31)

FANCF (11p14.3) FANCG (9p13.3) FANCL (2p16.1) FAS (10q23.31) FBXO11 (2p16.3) FBXW7 (4q31.3) FCRL4 (1q23.1) FEV (2q35) FGF10 (5p12) FGF14 (13q33.1) FGF19 (11q13.3) FGF23 (12p13.32) FGF3 (11q13.3) FGF4 (11q13.3) FGF6 (12p13.32) FGFR1 (8p11.23) FGFR1OP (6q27) FGFR2 (10q26.13) FGFR3 (4p16.3) FGFR4 (5q35.2) FH (1q43)

FHIT (3p14.2)

FIP1L1 (4q12) FLCN (17p11.2) FLI1 (11q24.3) FLT1 (13q12.3) FLT3 (13q12.2) FLT4 (5q35.3) FNBP1 (9q34.11) FOXA1 (14q21.1) FOXL2 (3q22.3) FOXO1 (13q14.11) FOXO3 (6q21) FOXO4 (Xq13.1) FOXP1 (3p13) FSTL3 (19p13.3) FUBP1 (1p31.1) FUS (16p11.2)

GAS7 (17p13.1) GATA1 (Xp11.23) GATA2 (3q21.3) GATA3 (10p14) GID4 (17p11.2) GMPS (3q25.31) GNA11 (19p13.3) GNA13 (17q24.1) GNAQ (9q21.2) GNAS (20q13.32) GOLGA5 (14q32.12) GOPC (6q22.1) GPC3 (Xq26.2) GPHN (14q23.3-24.1) GRIN2A (16p13.2) GSK3B (3q13.33)

H3-3A (1q42.12) H3-3B (17q25.1) H3C2 (6p22.2) H4C9 (6p22.1) HERPUD1 (16q13) HEY1 (8q21.13) HGF (7q21.11) HIP1 (7q11.23) HLF (17q22) HMGA1 (6p21.31) HMGA2 (12q14.3) HNF1A (12q24.31)

HNRNPA2B1 (7p15.2) HOOK3 (8p11.21) HOXA11 (7p15.2) HOXA13 (7p15.2) HOXA9 (7p15.2) HOXC11 (12q13.13) HOXC13 (12q13.13) HOXD11 (2q31.1) HOXD13 (2q31.1) HRAS (11p15.5)

HSP90AA1 (14q32.31) HSP90AB1 (6p21.1) IDH1 (2q34)

IDH2 (15q26.1) IGF1R (15q26.3) IKBKE (1q32.1) IKZF1 (7p12.2) IL2 (4q27) IL21R (16p12.1) IL6ST (5q11.2) IL7R (5p13.2) INHBA (7p14.1) IRF4 (6p25.3) IRS2 (13q34) ITK (5q33.3) JAK1 (1p31.3) JAK2 (9p24.1) JAK3 (19p13.11)

JAZF1 (7p15.2-15.1) JUN (1p32.1) KAT6A (8p11.21) KAT6B (10q22.2) KCNJ5 (11q24.3) KDM5A (12p13.33) KDM5C (Xp11.22) KDM6A (Xp11.3) KDR (4q12)

KDSR (18q21.33) KEAP1 (19p13.2) KIAA1549 (7q34) KIF5B (10p11.22) KIT (4q12)

KLF4 (9q31.2) KLHL6 (3q27.1) KLK2 (19q13.33) KMT2A (11q23.3) KMT2C (7q36.1) KMT2D (12q13.12)

KNL1 (15q15.1) KRAS (12p12.1) KTN1 (14q22.3) LASP1 (17q12) LCK (1p35.2) LCP1 (13q14.13)

LGR5 (12q21.1) LHFPL6 (13q13.3- 14.11)

LIFR (5p13.1) LMO1 (11p15.4) LMO2 (11p13) LPP (3q27.3-28) LRIG3 (12q14.1)

LRP1B (2q22.1-22.2) LYL1 (19p13.13) MAF (16q23.2) MAFB (20q12) MALT1 (18q21.32) MAML2 (11q21) MAP2K1 (15q22.31) MAP2K2 (19p13.3) MAP2K4 (17p12) MAP3K1 (5q11.2) MAX (14q23.3) MCL1 (1q21.2) MDM2 (12q15) MDM4 (1q32.1) MDS2 (1p36.11) MECOM (3q26.2) MED12 (Xq13.1) MEF2B (19p13.11) MEN1 (11q13.1) MET (7q31.2)

MITF (3p13) MLF1 (3q25.32) MLH1 (3p22.2) MLLT1 (19p13.3)

MLLT10 (10p12.31) MLLT11 (1q21.3) MLLT3 (9p21.3) MLLT6 (17q12) MN1 (22q12.1) MNX1 (7q36.3) MPL (1p34.2)

MRE11 (11q21) MRTFA (22q13.1- 13.2)

MSH2 (2p21-16.3) MSH6 (2p16.3) MSI2 (17q22) MSN (Xq12) MTCP1 (Xq28) MTOR (1p36.22) MUC1 (1q22) MUTYH (1p34.1) MYB (6q23.3) MYC (8q24.21) MYCL (1p34.2) MYCN (2p24.3) MYD88 (3p22.2) MYH11 (16p13.11) MYH9 (22q12.3) NACA (12q13.3) NBN (8q21.3)

NCKIPSD (3p21.31) NCOA1 (2p23.3) NCOA2 (8q13.3) NCOA4 (10q11.22) NDRG1 (8q24.22) NF1 (17q11.2)

NF2 (22q12.2) NFE2L2 (2q31.2) NFIB (9p23-22.3) NFKB2 (10q24.32) NFKBIA (14q13.2)

NIN (14q22.1) NKX2-1 (14q13.3) NONO (Xq13.1) NOTCH1 (9q34.3) NOTCH2 (1p12) NPM1 (5q35.1) NR4A3 (9q31.1) NRAS (1p13.2) NSD1 (5q35.3)

NSD2 (4p16.3)

NSD3 (8p11.23) NT5C2 (10q24.32- 24.33)

NTRK1 (1q23.1) NTRK2 (9q21.33) NTRK3 (15q25.3) NUMA1 (11q13.4) NUP214 (9q34.13) NUP93 (16q13) NUP98 (11p15.4) NUTM1 (15q14) NUTM2B (10q22.3) OLIG2 (21q22.11) OMD (9q22.31) P2RY8 (Xp22.33) PAFAH1B2 (11q23.3) PAK3 (Xq23)

PALB2 (16p12.2) PATZ1 (22q12.2) PAX3 (2q36.1) PAX5 (9p13.2) PAX7 (1p36.13) PAX8 (2q14.1) PBRM1 (3p21.1) PBX1 (1q23.3) PCM1 (8p22) PCSK7 (11q23.3) PDCD1 (2q37.3)

PDCD1LG2 (9p24.1) PDE4DIP (1q21.2) PDGFB (22q13.1) PDGFRA (4q12) PDGFRB (5q32) PDK1 (2q31.1) PER1 (17p13.1) PHF6 (Xq26.2) PHOX2B (4p13) PICALM (11q14.2) PIK3CA (3q26.32) PIK3CG (7q22.3) PIK3R1 (5q13.1) PIK3R2 (19p13.11) PIM1 (6p21.2) PLAG1 (8q12.1) PML (15q24.1) PMS1 (2q32.2) PMS2 (7p22.1) POLE (12q24.33) POT1 (7q31.33) POU2AF1 (11q23.1) POU5F1 (6p21.33) PPARG (3p25.2) PPP2R1A (19q13.41) PRCC (1q23.1) PRDM1 (6q21) PRDM16 (1p36.32) PRF1 (10q22.1) PRKAR1A (17q24.2) PRKDC (8q11.21) PRRX1 (1q24.2) PSIP1 (9p22.3) PTCH1 (9q22.32) PTEN (10q23.31) PTPN11 (12q24.13)

PTPRC (1q31.3-32.1)

RABEP1 (17p13.2)

RAC1 (7p22.1) RAD21 (8q24.11) RAD50 (5q31.1) RAD51 (15q15.1) RAD51B (14q24.1)

RAF1 (3p25.2) RALGDS (9q34.13- 34.2)

RANBP17 (5q35.1) RAP1GDS1 (4q23) RARA (17q21.2) RB1 (13q14.2) RBM15 (1p13.3) RECQL4 (8q24.3) REL (2p16.1)

RET (10q11.21) RHOH (4p14) RICTOR (5p13.1) RMI2 (16p13.13) RNF213 (17q25.3) RNF43 (17q22) ROS1 (6q22.1) RPL10 (Xq28) RPL22 (1p36.31) RPL5 (1p22.1) RPN1 (3q21.3) RPTOR (17q25.3) RUNX1 (21q22.12) RUNX1T1 (8q21.3) SBDS (7q11.21) SDC4 (20q13.12) SDHAF2 (11q12.2) SDHB (1p36.13) SDHC (1q23.3) SDHD (11q23.1) SEPTIN5 (22q11.21) SEPTIN6 (Xq24) SEPTIN9 (17q25.3)

SET (9q34.11) SETBP1 (18q12.3) SETD2 (3p21.31) SF3B1 (2q33.1) SFPQ (1p34.3) SH2B3 (12q24.12) SH3GL1 (19p13.3) SLC34A2 (4p15.2) SLC45A3 (1q32.1) SMAD2 (18q21.1) SMAD4 (18q21.2) SMARCA4 (19p13.2)

SMARCB1 (22q11.23)

SMARCE1 (17q21.2)

SMO (7q32.1) SNX29 (16p13.13- 13.12)

SOCS1 (16p13.13) SOX10 (22q13.1) SOX2 (3q26.33) SPECC1 (17p11.2) SPEN (1p36.21-36.13) SPOP (17q21.33) SRC (20q11.23) SRGAP3 (3p25.3) SRSF2 (17q25.1) SRSF3 (6p21.31-21.2) SS18 (18q11.2) SS18L1 (20q13.33) SSX1 (Xp11.23) STAG2 (Xq25) STAT3 (17q21.2) STAT4 (2q32.2-32.3) STAT5B (17q21.2) STIL (1p33)

STK11 (19p13.3) SUFU (10q24.32) SUZ12 (17q11.2)

SYK (9q22.2) TAF15 (17q12) TAL1 (1p33) TAL2 (9q31.2)

TBL1XR1 (3q26.32) TCEA1 (8q11.23) TCF12 (15q21.3)

TCF3 (19p13.3) TCF7L2 (10q25.2- 25.3)

TCL1A (14q32.13) TENT5C (1p12) TERT (5p15.33) TET1 (10q21.3) TET2 (4q24)

TFE3 (Xp11.23) TFEB (6p21.1) TFG (3q12.2) TFPT (19q13.42) TFRC (3q29) TGFBR2 (3p24.1) THRAP3 (1p34.3) TLX1 (10q24.31) TLX3 (5q35.1)

TMPRSS2 (21q22.3) TNFAIP3 (6q23.3) TNFRSF14 (1p36.32) TNFRSF17 (16p13.13) TOP1 (20q12)

TP53 (17p13.1)

TPM3 (1q21.3) TPM4 (19p13.12- 13.11)

TPR (1q31.1) TRAF7 (16p13.3) TRIM26 (6p22.1) TRIM27 (6p22.1) TRIM33 (1p13.2) TRIP11 (14q32.12)

TRRAP (7q22.1) TSC1 (9q34.13) TSC2 (16p13.3) TSHR (14q31.1) TTL (2q14.1) U2AF1 (21q22.3) UBR5 (8q22.3) USP6 (17p13.2) VEGFA (6p21.1) VEGFB (11q13.1) VHL (3p25.3) VTI1A (10q25.2) WAS (Xp11.23) WDCP (2p23.3) WIF1 (12q14.3) WRN (8p12) WT1 (11p13) WWTR1 (3q25.1) XPA (9q22.33) XPC (3p25.1) XPO1 (2p15)

YWHAE (17p13.3)

ZBTB16 (11q23.2) ZMYM2 (13q12.11) ZNF217 (20q13.2) ZNF331 (19q13.42) ZNF384 (12p13.31) ZNF521 (18q11.2) ZNF703 (8p11.23) ZRSR2 (Xp22.2)

Supplementary Table 2 Genes

ABL1 ABRAXAS1 ACVR1

AIP AKT1 AKT2

AKT3 ALK AMER1 APC AR ARAF

ARID1A ARID1B ARID2 ASXL1 ATM ATR ATRX AURKB B2M BAP1 BARD1 BCL2 BCOR BCORL1 BLM BMPR1A BRAF BRCA1 BRCA2 BRIP1 CARD11 CBFB CCND1 CCND2 CCND3 CCNE1 CD274 CDC73 CDH1 CDK12 CDK4 CDK6 CDKN1B CDKN1C

CDKN2A CDKN2B CHEK1 CHEK2 CIC CLYBL CREBBP CRKL CSF1R CTNNB1 CYLD DAXX DDR2 DICER1 DNMT3A EGFR EME1 EPCAM EP300 EPHA2 ERBB2 ERBB3 ERBB4 ERCC2 ESR1 EZH2 FANCA FANCB FANCC FANCD2 FANCE FANCF FANCG FANCI FANCL FANCM FAS FAT1 FBXW7 FGF10

FGF19 FGF3 FGF4 FGFR1 FGFR2 FGFR3 FGFR4 FH FLCN FLT1 FLT3 FLT4 FOXL2 FUBP1 FYN GATA3 GLI2 GNA11 GNA13 GNAQ GNAS H3F3A H3F3B

HIST1H3B HNF1A HRAS

ID2 IDH1 IDH2 JAK1 JAK2 JAK3 KAT6A KDM5C KDM6A KDR KIT KLLN KMT2A KMT2C

KMT2D KRAS LCK LYN LZTR1 MAP2K1 MAP2K2 MAP2K4 MAP3K1 MAX MDM2 MDM4 MEF2B MEN1 MET MITF MLH1 MLH3 MPL MRE11 MSH2 MSH3 MSH6 MST1R MTOR MUTYH MYB MYC MYCL MYCN NBN NF1

NF2 NFKBIA NOTCH1 NPM1 NRAS NSD1 NTHL1 NTRK1

NTRK2 NTRK3 PALB2 PBRM1 PDCD1 PDCD1LG2 PDGFRA PDGFRB PER1 PHOX2B PIK3CA PIK3R1 PIM1 PMS1 PMS2 POLD1 POLE POT1 PPARG PPP2R1A PRDM1 PRKAR1A PRKDC PTCH1 PTEN PTPN11 RAD50 RAD51C RAD51D RAF1

RB1 RET RICTOR RNF43 ROS1 RSPO1 RSPO2 RSPO3 RUNX1 SDHA

SDHAF2 SDHB SDHC SDHD SETD2 SF3B1 SLX4 SMAD2 SMAD4 SMARCA4 SMARCB1 SMARCE1 SMO SOCS1 SPEN SPOP

SRC STK11 SUFU TERC TMEM127 TNFAIP3 TNFRSF14 TOP1 TOP2A TP53 TSC1 TSC2 U2AF1 UBR5 VHL

WRN WT1 YAP1 YES1 ZNRF3 ABL2 ACKR3 ACSL3 ACSL6

AFDN AFF1 AFF3 ARNT ATF1 ATIC ATP1A1 BCL11A BCL11B BCL2L11 BCL2L2 BCL3 BCL6 BCL9 BRD4 BTG1

C15orf65 CACNA1D CALR CAMTA1 CARS CASP8 CBL CCDC6 CD74 CD79A CDH11 CDK8 CDKN2C CDX2 CEBPA CHIC2 CLTCL1 CNBP COX6C CREB3L2 CRTC3 CTCF CTNNA1 DDIT3

DDX41 DDX6 DEK EBF1 ECT2L ELK4 EPHA3 ERCC1 ERCC3 ERG ETV1 ETV5 ETV6 EWSR1 EXT1 EXT2 EZR FAM46C FCRL4 FGF23 FGFR1OP FHIT FLI1 FNBP1 FOXA1 FOXO1 FOXO3 FOXP1 FSTL3 FUS GATA2 GID4 GMPS GRIN2A HEY1 HIST1H4I HLF HMGA2

HMGN2P46 HOOK3

HOXA11 HOXA13 HOXA9 HOXB13 HOXD13 HSP90AA1 HSP90AB1 IGF1R IKZF1 IL7R

IRF4 ITK JAZF1 JUN KAT6B KCNJ5 KDSR

KIAA1549 KIF5B KLF4 KLHL6 KLK2 LHFPL6 LIFR

LPP LRP1B MAF MAML2 MCL1 MDS2 MECOM MLF1 MLLT10 MLLT11 MLLT3 MN1 MSI2 MUC1 MYD88 NCOA2

NDRG1 NFIB NFKB2 NIN NKX2-1 NOTCH2 NR4A3 NUP214 NUP93 NUP98 NUTM1 OLIG2

PAFAH1B2 PAX3 PAX5 PAX8 PBX1 PCM1 PDE4DIP POU2AF1 PRCC PRRX1 PTPRC RAC1 RHOH RMI2 RPL22 RPN1 RUNX1T1 SBDS SDC4 SETBP1 SFPQ SLC34A2 SNX29 SOX10 SOX2 SPECC1 SPRED1 SRGAP3

SRSF2 SRSF3 STAT3 STAT5B STIL SUZ12 SYK TAF15 TBXT TCEA1 TCF7L2 TERT TET1 TFRC TGFBR1 TGFBR2 THRAP3 TPM3 TPM4 TRIM27 TRRAP TSHR USP6 VTI1A WDCP WISP3 WWTR1 XPC YWHAE ZBTB16 ZNF217 ZNF331 ZNF384 ZNF521 ABCB11 ACD ACVR1B ADGRA2 AJUBA ALOX12B

ANKRD26 APLNR ARFRP1 ARHGAP35 ARHGEF12 ARID5B ASPSCR1 ASXL2 AURKA AXIN1 AXIN2 AXL BCL10 BCL2L1 BCL2L12 BIRC3 BRD3 BTG2

BTK BUB1B CBFA2T3 CBLB CD22 CD70 CD79B CDH23 CDKN1A CHD2 CHD4 CHN1 CIITA CNOT3 CREB1 CREB3L1 CRLF2 CRTC1 CSF3R CTLA4 CUL3 CUL4A

CUX1 CXCR4 CYP17A1 CYP2D6 DDB2 DDR1 DDX3X DIS3 DIS3L2 DKC1 DMC1 DNA2 DNAJB1 DOT1L EED EGLN1 EIF1AX EIF4A2 ELF3 ELOC EME2 EML4 EMSY EPHA5 EPHA7 EPHB1 EPHB4 ERC1 ERCC4 ERCC5 ERCC6 EREG ERRFI1 ETS1 ETV4 EXO1 FAT3 FBXO11 FEN1 FGF12

FGF14 FGF6 FIP1L1 FOXO4 FRS2 GABRA6 GALNT12 GATA1 GATA4 GATA6 GEN1 GLI1 GOPC GPC3 GPS2 GREM1 GRM3 GSK3B H2AFX HDAC1 HGF HIF1A HIST1H3C HLA-A HRG HSD3B1 ID3 IFNGR1 IGF2 IKBKE INHBA INPP4B IRF1

IRF2 IRS2 KDM5A KEAP1 KEL KIF1B LDLR

LIG1 LMNA LMO1 LMO2 LTK MAFB MAGI2 MALT1 MAP3K13 MAPK1 MAPK3 MBD4 MDC1 MED12 MERTK MGA MGMT MKNK1 MTAP MTCP1 MUS81 MYH11 MYH9 NCOA3 NCOA4 NCOR1 NFE2L2 NFKBIE NONO NOTCH3 NSD2 NSD3 NT5C2 P2RY8 PAK1 PAK3 PARP1 PARP2 PARP3 PAX7

PDGFB PDK1 PHF6 PIK3C2B PIK3C2G PIK3CB PIK3CD PIK3CG PIK3R2 PLAG1 PLCG2 PML POLD2 POLD3 POLD4 POLH POLQ PPM1D PPP2R2A PPP6C PREX2 PRF1 PRKACA PRKCH PRKCI PRKN PRSS1 PRSS8 PTCH2 PTK2B PTPN22 PTPRD PTPRO PTPRT QKI RAD21 RAD51 RAD51B RAD52 RAD54B

RAD54L RANBP2 RARA RASA1 RBBP8 RBM10 RCAN1 RECQL4 REL RELA RHEB RHOA RINT1 RIT1 RPA1 RPA2 RPA3 RPA4 RPL10 RPL5 RPTOR RRAS2 SEM1 SERPINB3 SET

SGK1 SH2B3 SLIT2 SMAD3 SMARCA1 SMC3 SNCAIP SOS1 SOX9 SPTA1 SS18 SSBP1 STAG2 STAT4 STAT6

TAF1 TAL1 TAL2 TBX3 TCF3 TEK TERF2IP TET2 TFE3 TFEB TFG TIPARP TLX3 TNF TOP3A TOP3B TRAF3 TRAF7 TSHZ3 TYK2 TYRO3 UBE2T VEGFA WAS XPA XPO1 XRCC1 XRCC2 XRCC3 ZBTB2 ZFHX3 ZNF703 ZRSR2

Supplementary Figure 1


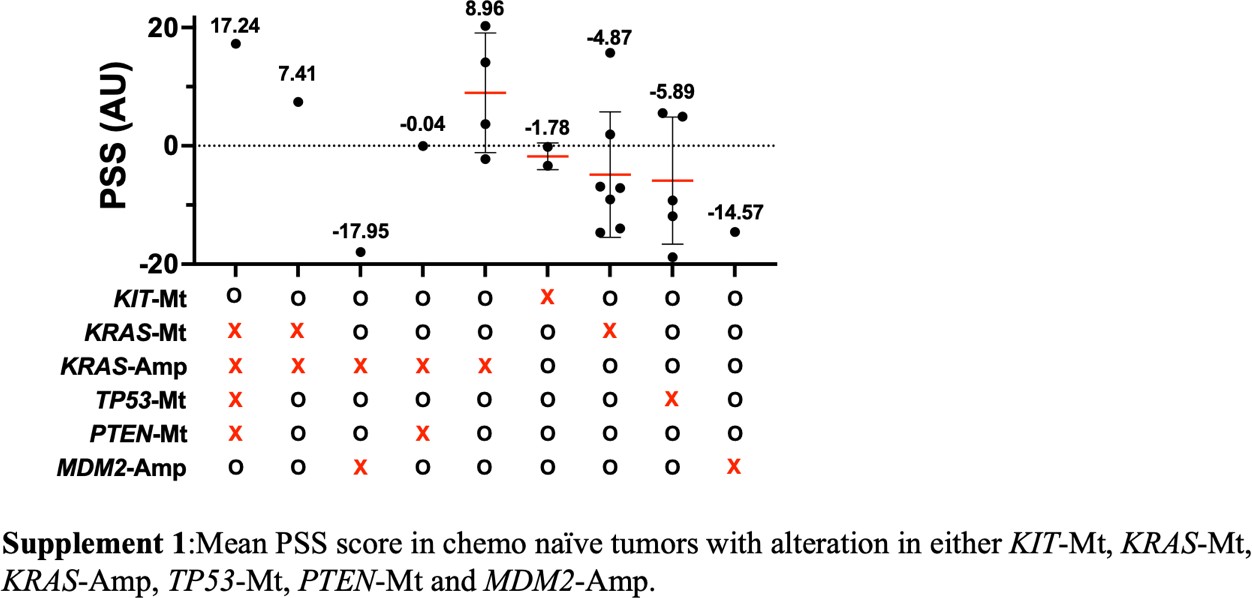


Supplementary Figure 2
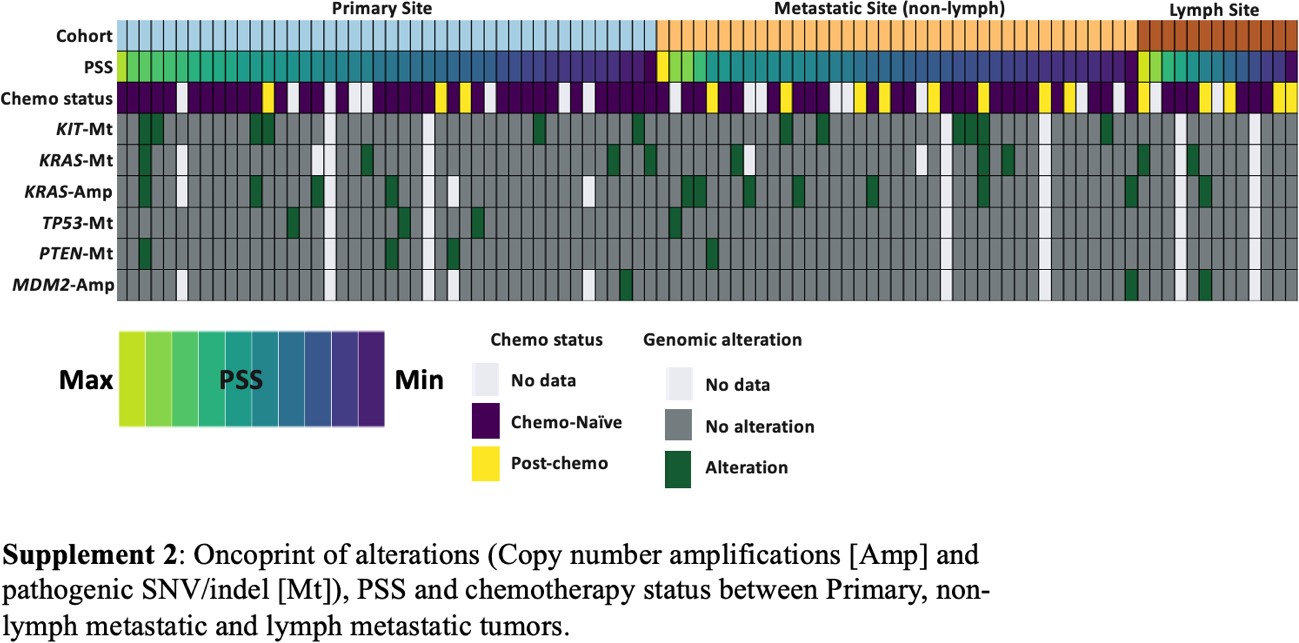


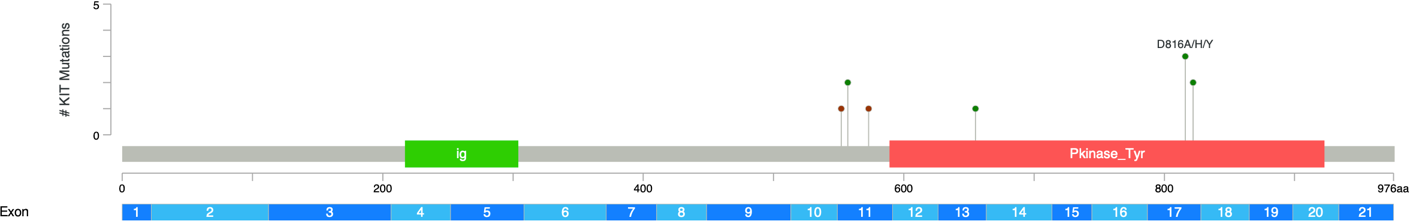


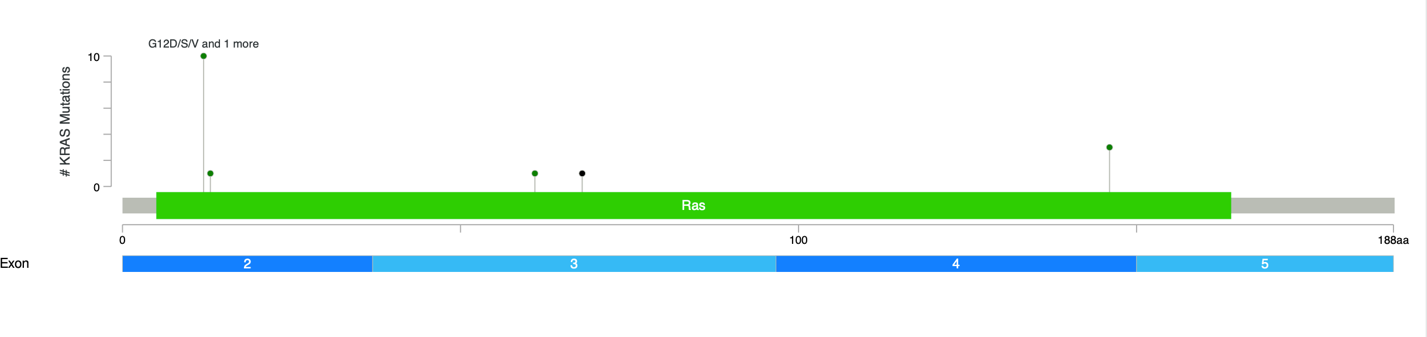

Supplement: Supplementary file 1 — Supplemental Material [file 41698_2024_727_MOESM1_ESM.docx]
